# Chrysin Reduces Oxidative Stress but Does Not Affect Polyol Pathway in the Lenses of Type 1 Diabetic Rats

**DOI:** 10.3390/antiox9020160

**Published:** 2020-02-16

**Authors:** Weronika Wojnar, Maria Zych, Sławomir Borymski, Ilona Kaczmarczyk-Sedlak

**Affiliations:** 1 Department of Pharmacognosy and Phytochemistry, Faculty of Pharmaceutical Sciences in Sosnowiec, Medical University of Silesia, Katowice, Jagiellońska 4, 41-200 Sosnowiec, Poland; mzych@sum.edu.pl (M.Z.); isedlak@sum.edu.pl (I.K.-S.); 2 Faculty of Natural Sciences, Institute of Biology, Biotechnology and Environmental Protection, University of Silesia, Jagiellońska 28, 40-032 Katowice, Poland; slawomir.borymski@us.edu.pl

**Keywords:** type 1 diabetes, chrysin, lens, rats, oxidative stress, oxidative damage

## Abstract

Prolonged hyperglycemia is one of the main causes of reactive oxygen species and free radicals generation in diabetes which may affect various organs, including the eye. Oxidative damage to proteins and lipids in the eye lens could lead to cataract formation. To cope with oxidative stress, the endogenous antioxidative system may be supported by the supplementation of exogenous antioxidants. The aim of this study was to evaluate the effect of chrysin, a natural flavonoid, on oxidative stress and polyol pathway-related markers in the lenses of streptozotocin-induced type 1 male diabetic rats. Chrysin at doses of 50 and 100 mg/kg was administered by gavage for 28 days. This treatment resulted in a decrease in antioxidative enzymes activity and oxidative stress index. Moreover, chrysin administration elevated the reduced glutathione level in the lenses. A decrease in the markers linked to oxidative damage to proteins and lipids in the lenses was noted, especially after treatment with 50 mg/kg of chrysin. Neither of the chrysin doses affected glycemia-related markers in the serum or altered parameters related to the polyol pathway and advanced glycation end-products level in the lenses of diabetic rats. Upon obtaining results, it can be concluded that chrysin reveals antioxidative activity in the lenses but shows no antihyperglycemic or antiglycation properties.

## 1. Introduction

The term “oxidative stress”, describing an oxidative damage to the cells or organs, was proposed in 1985 and was originally defined as “a disturbance in the prooxidant-antioxidant balance in favor of the former” [1]. There are many types of prooxidants, such as free radicals or reactive oxygen species (ROS). Free radicals are particles with one or more unpaired electrons and ROS are reactive compounds containing oxygen. Some of ROS, e.g., superoxide anion radical, hydroxyl radical or peroxyl radical, may be classed as free radicals as they possess unpaired electrons. Other ROS, such as hydrogen peroxide or ozone, are not free radicals, but they could also pose as prooxidants [2,3,4]. Under normal physiological conditions, ROS are produced mainly in the mitochondrial respiratory chain. In smaller amounts they are also generated in endoplasmic reticulum and peroxisomes, as well as during “respiratory burst” with involvement of the NADPH oxidase complex. Moreover, ROS may arise from the autooxidation of endogenous particles such as epinephrine [3,5]. These endogenous ROS serve as signaling molecules in many physiological processes and cellular defense, and their level is strictly controlled by appropriate antioxidative mechanisms [3,4,6].

In diabetes, excessive free radicals and ROS are generated through various pathways as a result of hyperglycemia. Glucose in enediol form may be oxidized to an enediol radical anion which is transformed into ketoaldehydes and superoxide anion radicals. The latter may lead to the production of highly reactive hydroxyl radicals or peroxynitrite radicals. The excess of glucose also promotes peroxidation of lipids in low density lipoproteins, that can consequently lead to free radicals production. Moreover, in hyperglycemic conditions glucose may interact with proteins producing advanced glycation end-products (AGEs), which could promote free radicals generation [7,8]. Overproduced ROS interact with numerous structures, including proteins and lipids, leading to oxidative damage [2,4]. Oxidative stress resulting from hyperglycemia is also a trigger for diabetic complications affecting many organs including the eye [7,9]. There are many types of ocular complications in diabetic patients. Such complications as glaucoma, diabetic retinopathy, keratopathy, and the inflammation of eyelids or cataract may be included. Cataract is a disease characterized by lens opacity. It causes distortions like overall worsening of the vision, blurriness, double or distorted vision, increased glare and halo around lights, changes in color vision and, eventually, it may lead to blindness [10,11,12]. It is widely described that cataract is about 2–4 times more frequent and occurs earlier in diabetic patients than in healthy people of the same age. For instance, cataract formation is a problem in children and adolescents with type 1 diabetes [11,12,13,14,15]. It is estimated that one-fifth of cataract removal surgeries are carried out in diabetic patients. Although nowadays cataract surgery is a safe procedure, diabetic patients are at higher risk of peri- and postoperative complications including higher probability of anterior segment inflammation and diabetic retinopathy or diabetic macular edema development [13,16].

Oxidative stress is said to be one of the major factors in cataract formation. In order to cope with free radicals and ROS, the body possesses an antioxidative system composed of enzymatic and non-enzymatic antioxidants [2,17]. However, when the endogenous antioxidants are insufficient to neutralize the oxidants, exogenous antioxidants can be delivered, for example with a diet or dietary supplements, to increase the antioxidative abilities of the body. Moreover, there is more and more evidence suggesting that changing lifestyle and nutrition, including appropriate supplementation, may prevent cataract formation [11]. Among such exogenous antioxidants which can be supplemented, there are vitamins, carotenoids or polyphenols, including flavonoids [2]. Chrysin (5,7-di-OH-flavone), a naturally occurring flavonoid belonging to the flavone subgroup, can be mainly found in the blue passion flower (*Passiflora caerulea* L.) and in products made by honeybees—propolis and honey. It is also present in the roots of skullcap (*Scutellaria* sp. L.) and in the pearl oyster mushroom (*Pleurotus ostreatus* (Jacq. ex Fr.) P.Kumm). This flavonoid reveals many beneficial physiological effects, including antioxidative properties [18,19]. There are also reports on its positive effect on ocular structures, including the lenses [20,21,22,23,24,25], but none of these studies were conducted in vivo in diabetic rats. Therefore, our goal was to determine if chrysin administered by intragastric tube may counteract the diabetes-induced changes in the markers linked to oxidative stress in the lenses of rats. 

## 2. Materials and Methods 

### 2.1. Animals, Drugs and Diabetes Induction

The study was conducted on three-month-old male Wistar rats provided by the Centre of Experimental Medicine at the Medical University of Silesia in Katowice. All procedures were approved by the Local Ethics Committee in Katowice, Poland (approvals no. 36/2015 and 114/2015). During the whole experiment the animals were fed with a standard laboratory chow (Labofeed B, Wytwórnia Pasz “Morawski”, Kcynia, Poland) and had unlimited water supply. The rats were kept in standard plastic cages (4–5 rats per cage) under an equal photoperiod (12 h of light and 12 h of dark). All conditions met the European Union guidelines (directive 2010/63/EU).

The rats were divided into the following groups:

- Healthy control rats—group C
- Diabetic control rats—group DM
- Diabetic rats treated by gavage with chrysin at a dose of 50 mg/kg—group CHR50
- Diabetic rats treated by gavage with chrysin at a dose of 100 mg/kg—group CHR100

Diabetes in the DM, CHR50 and CHR100 groups of rats was induced by a single intraperitoneal injection of 60 mg/kg of streptozotocin (STZ, Cayman Chemical, Ann Arbor, MI, USA) dissolved in 0.1 M citric buffer (pH 4.5). The rats from the C group were injected only with 0.1 M citric buffer (pH 4.5). Streptozotocin solution was prepared freshly before injections by dissolving 60 mg of STZ in 1 mL of citric buffer. The volume of injected solution was adjusted to every rat according to its current body mass (1 mL of solution per 1 kg of body mass). Two weeks after STZ injection, the non-fasting glucose level from the blood obtained from the tail vessels was measured with the use of a MicroDot glucometer equipped with test strips (Cambridge Sensor USA, Plainfield, IL, USA). If the blood glucose level exceeded 200 mg/dL, the animals were classified as diabetic and subjected to further steps of the study. Chrysin (Sigma-Aldrich, St. Louis, MO, USA) suspended in water was administered once a day via intragastric tube from the day of diabetes confirmation for 28 days. Based on literature data and the fact that chrysin reveals low yet dose-dependent bioavailability, two doses of this flavone (50 mg/kg and 100 mg/kg) were chosen for this study [18,26,27,28]. Chrysin suspensions were prepared daily, directly before administration, by suspending 50 or 100 mg of chrysin in 1 mL of water. The volume of administered suspension was adjusted to the current body mass of each rat (1 mL of suspension per 1 kg of body mass). The C and DM rats received water by gavage in a volume corresponding to their current body mass (1 mL of water per 1 kg of body mass). This research shares the control C and DM groups with our previously presented studies [29,30,31,32].

The next day after administration of the last dose of chrysin, non-fasted rats fully anesthetized with a single intraperitoneal injection of mixture composed of ketamine and xylazine (Ketamina 10%, Biowet Puławy Sp. z o. o., Puławy, Poland and Xylapan, Vetoquinol Biowet, Gorzów Wlkp., Poland; 87.5 mg/kg of ketamine and 12.5 mg/kg of xylazine was injected) were euthanized by cardiac perfusion. The total volume of the blood collected from the heart was centrifuged in order to receive serum for biochemical analyses. Subsequently, the eyeballs were excised from the eye sockets in order to extract the lenses. The lenses were weighted and homogenized in phosphate buffered saline, pH 7.4 (10% v/w) and divided. Part of the homogenate was centrifuged at 10,000× *g*, (15 min, +4 °C) and used for further analyses. In the non-centrifuged part of the homogenate, the levels of total protein and malondialdehyde were evaluated. The spectrophotometric and ELISA assays conducted in this study were measured in a Tecan Infinite M200 PRO plate reader with Magellan 7.2 software (Tecan Austria, Grödig, Austria).

### 2.2. Evaluation of Glycemia-Related Parameters in the Serum

The serological non-fasting glucose level and the fructosamine level were evaluated with BioSystems kits (BioSystems S.A., Barcelona, Spain), while insulin level was measured with a Mercodia Ultrasensitive Rat Insulin ELISA kit (Mercodia AB, Uppsala, Sweden). 

### 2.3. Evaluation of Protein Level, Antioxidants Level and Antioxidative Enzymes Activity in the Lenses

The total protein and soluble protein levels were evaluated according to Lowry’s method [33] in total and centrifuged homogenates, respectively. 

Superoxide dismutase (SOD), catalase (CAT), glutathione peroxidase (GPx) and glutathione reductase (GR) activities were measured with Cayman kits (Cayman Chemical, Ann Arbor, MI, USA). The activity of glucose-6-phosphate dehydrogenase (G6PD) was assessed with a Pointe Sci. kit (Pointe Scientific, Inc., Canton, MI, USA). Reduced (GSH), oxidized (GSSG) and total (total GSH) glutathione levels were determined with the Cayman kit. The assay proposed by Jagota and Dani [34] was used in order to evaluate the level of vitamin C. 

### 2.4. Evaluation of Oxidative Damage Markers Level in the Lenses

To evaluate the oxidative damage to lipids, the level of malondialdehyde (MDA) in total homogenate was assessed, according to the Ohkawa et al. method [35]. Two markers of protein oxidation were assayed: protein carbonyl groups (PCG) level was evaluated by an OxiSelect™ kit (Cell Biolabs, San Diego, CA, USA), while advanced oxidation protein products (AOPP) level was estimated with the Witko–Sarsat method [36].

### 2.5. Evaluation of Total Oxidatant/Antioxidant Status in the Lenses

Total antioxidant response (TAR) was evaluated according to the Erel method [37]. Another Erel’s protocol was used in order to estimate total oxidative status (TOS) [38]. TAR and TOS values were used for the oxidative stress index (OSI) calculation [39]:

$$\mathrm{OSI}=\frac{\mathrm{TOS}}{\mathrm{TAR}\times 100}$$

### 2.6. Evaluation of Polyol Pathway Markers and AGEs in the Lenses

Glucose and fructose levels in the lenses were examined with BioSystems kits. The sorbitol level was evaluated with BioAssays EnzyChrome kit (BioAssay Systems, Hayward, CA, USA). The activity of aldose reductase (AR) was tested according to the method presented by Patel et al. [40] and the activity of sorbitol dehydrogenase (SDH) was evaluated with a BioAssays QuantiChrom kit (BioAssay Systems, Hayward, CA, USA). The advanced glycation end-products (AGEs) level was assessed with an OxiSelect ELISA kit (Cell Biolabs, Inc., San Diego, CA, USA).

### 2.7. Statistical Analyses

The results obtained during the experiment were subjected to a one-way ANOVA test followed by a Fisher’s Least Significant Difference (LSD) post-hoc test. The differences were considered statistically significant if *p* < 0.05. Only results for the rats in which type 1 diabetes occurred were used for the analyses. As not all rats survived the experiment period, due to a severe hyperglycemia and overall exhaustion, the final number of the rats was as follows: C group: *n* = 9, DM group: *n* = 8, CHR50 group: *n* = 9, CHR100 group: *n* = 7. All data are presented as arithmetical mean ± standard deviation (SD). The means, SD and ANOVA with the post-hoc test were calculated in the Statistica 13 software (StatSoft, Kraków, Poland). All the results obtained for the lenses were also subjected to principal component analysis (PCA), based on correlation matrix. Spearman’s rs correlations for the results obtained from the serum and the lenses were also calculated (Appendix A). The PCA and correlations were calculated using the PAST 3.20 Software [41]. Additionally, the PCA output was subjected to a MANOVA in the Statistica 13 software (Appendix B).

## 3. Results

### 3.1. Effect of Chrysin on Glycemia-Related Markers in the Serum

Administration of chrysin at the doses of 50 and 100 mg/kg for 28 days did not reduce the non-fasting glucose level in the serum, which was elevated after diabetes induction in the diabetic control (DM) rats. The glucose level remained higher in the CHR50 and CHR100 groups of rats than in the control C rats. However, the level of glucose in the serum of the CHR100 rats was significantly lower than in the serum of the CHR50 rats. The fructosamine level in the serum of the DM rats was significantly higher than in the serum of the C rats and neither of the chrysin doses affected this parameter after 28 days of treatment. Treatment with chrysin at both the doses also did not alter the insulin level in the serum, which was significantly reduced in the DM rats as compared to the C rats (Table 1).

### 3.2. Effect of Chrysin on Protein Level, Antioxidants Level and Antioxidative Enzymes Activity in the Lenses

**Figure 1:**
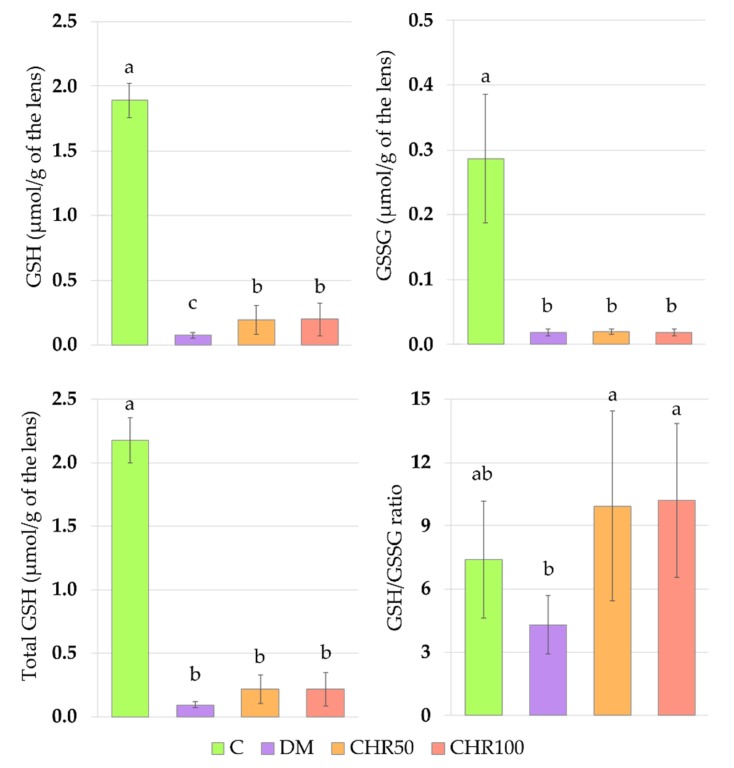
Effect of chrysin on the reduced, oxidized and total glutathione levels and GSH/GSSG ratio in the lenses of the type 1 diabetic rats. Results are presented as arithmetical means ± SD. The letters (a–c) above the bars indicate statistical significance (one-way ANOVA followed by Fisher’s LSD test). The same letter(s) above the bars indicate no significant differences of values in measured parameters at *p* < 0.05. C—control, non-diabetic rats; DM—control, type 1 diabetic rats; CHR50—type 1 diabetic rats treated by gavage with chrysin at a dose of 50 mg/kg for 28 days; CHR100—type 1 diabetic rats treated by gavage with chrysin at a dose of 100 mg/kg for 28 days; GSH—reduced glutathione; GSSG—oxidized glutathione; Total GSH—total glutathione.

Administration of chrysin to the diabetic rats resulted in an increase of reduced glutathione (GSH) level in the lenses, compared to the DM control rats, in which the GSH level was significantly reduced in comparison with the C rats. The GSH level in the lenses of the CHR50 and CHR100 rats remained significantly lower when compared with the C rats. Even though the GSSG level in the lenses of chrysin-treated rats did not change compared to the DM rats, the GSH/GSSG ratio in these groups of rats was significantly higher than in the DM animals and did not differ from the value noted in the C rats. The total GSH level (a sum of reduced and oxidized glutathione forms) was significantly lower in the lenses of the DM rats than in the lenses of the non-diabetic C rats. Administration of both chrysin doses to diabetic rats did not elevate the total GSH level in their lenses compared to the lenses of the DM group of rats. The total GSH level in the lenses of the CHR50 and CHR100 rats remained significantly lower than the lenses of the C rats (Figure 1). The activity of glutathione peroxidase (GPx) in the lenses of the CHR50 rats was significantly lower than in the lenses of both the DM and C rat groups. Although the GPx activity in the lenses of the CHR100 rats was not different from the activity of GPx in the lenses of the CHR50 rats, the CHR100 GPx activity was not lower than in the lenses of the C or DM rats. No statistically significant differences in the activity of glutathione reductase (GR) or glucose-6-phosphate dehydrogenase (G6PD) were noted in the lenses of the tested animals (Table 2).

There were no significant differences in the lenticular total protein level between all tested groups of rats. On the other hand, the soluble protein level in the lenses of diabetic rats treated with both doses of chrysin was significantly higher than in the untreated DM rats. The vitamin C level in the lenses of CHR50 rats was lower than in the lenses of the control healthy C rats and was comparable to the level observed in the lenses of the DM rats. There were no differences in the vitamin C level in the lenses of the CHR100 rats and the level of vitamin C in the lenses of the remaining rat groups (Table 2).

**Figure 2:**
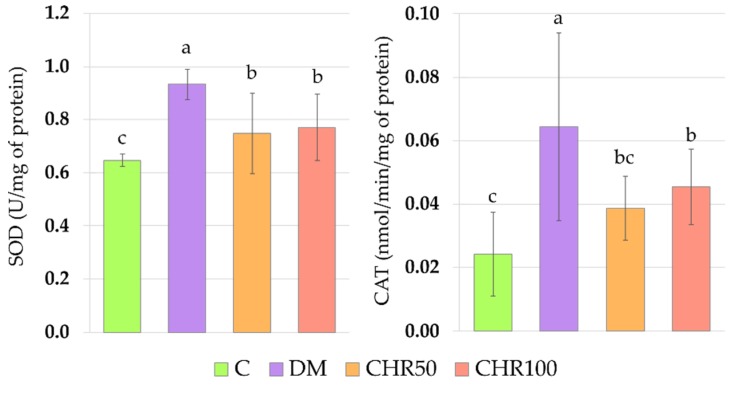
Effect of chrysin on the activity of antioxidative enzymes in the lenses of the type 1 diabetic rats. Results are presented as arithmetical means ± SD. The letters (a–c) above the bars indicate statistical significance (one-way ANOVA followed by Fisher’s LSD test). The same letter(s) above the bars indicate no significant differences of values in measured parameters at *p* < 0.05. C—control, non-diabetic rats; DM—control, type 1 diabetic rats; CHR50—type 1 diabetic rats treated by gavage with chrysin at a dose of 50 mg/kg for 28 days; CHR100—type 1 diabetic rats treated by gavage with chrysin at a dose of 100 mg/kg for 28 days; SOD—superoxide dismutase; CAT—catalase.

The activity of superoxide dismutase (SOD) in the lenses of CHR50 and CHR100 rats was significantly lower than in the lenses of DM rats, but remained significantly higher than in the lenses of the C rats. The administration of chrysin at a dose of 50 mg/kg to the diabetic rats resulted in a significant decrease of catalase (CAT) activity, in comparison with the DM rats. In this group of rats, the activity of CAT was not significantly different from the activity of CAT in the lenses of the control C rats. The higher dose of chrysin (100 mg/kg) administered to the diabetic rats for 28 days also resulted in a decrease in CAT activity in the lenses in comparison with the DM rats, but this parameter was still significantly higher than in the lenses of the C rats (Figure 2).

### 3.3. Effect of Chrysin on Oxidative Damage Markers Level in the Lenses

**Figure 3:**
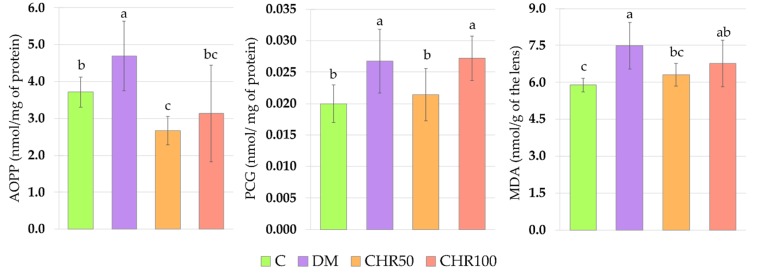
Effect of chrysin on oxidative damage markers level in the lenses of the type 1 diabetic rats. Results are presented as arithmetical means ± SD. The letters (a–c) above the bars indicate statistical significances (one-way ANOVA followed by Fisher’s LSD test). The same letter(s) above the bars indicate no significant differences of values in measured parameters at *p* < 0.05. C—control, non-diabetic rats; DM—control, type 1 diabetic rats; CHR50—type 1 diabetic rats treated by gavage with chrysin at a dose of 50 mg/kg for 28 days; CHR100—type 1 diabetic rats treated by gavage with chrysin at a dose of 100 mg/kg for 28 days; AOPP—advanced oxidation protein products; PCG—protein carbonyl groups; MDA—malondialdehyde.

The induction of diabetes resulted in a significant increase in the levels of all tested oxidative damage markers in the lenses of diabetic rats. The administration of chrysin at a dose of 50 mg/kg for 28 days to the diabetic rats resulted in a decrease in the lenses of both the markers describing oxidative damage to the proteins—advanced oxidation protein products (AOPP) and protein carbonyl groups (PCG), as well as in a decrease in lipids oxidation marker level—malondialdehyde (MDA). The AOPP level in the lenses of the CHR50 rats was even lower than in the control C rats. The higher dose of chrysin (100 mg/kg) administered to the diabetic rats reduced only the AOPP level in the lenses. The MDA level in the lenses of the CHR100 rats was not lower than in the DM rats and was higher than in the C rats, but it was not significantly different from the MDA level in the lenses of the CHR50 rats (Figure 3).

### 3.4. Effect of Chrysin on Total Oxidatant/Antioxidant Status in the Lenses

**Figure 4:**
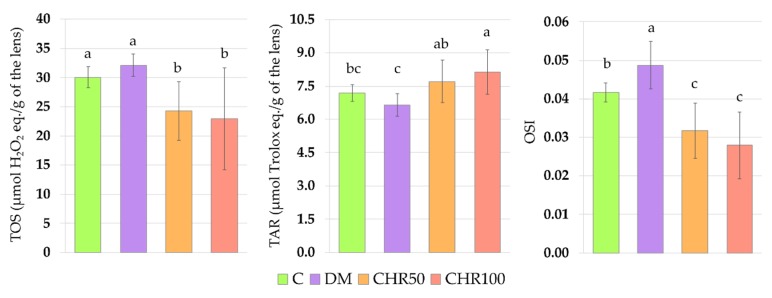
Effect of chrysin on total oxidative status, total antioxidant response and oxidative stress index in the lenses of the type 1 diabetic rats. Results are presented as arithmetical means ± SD. The letters (a–c) above the bars indicate statistical significances (one-way ANOVA followed by Fisher’s LSD test). The same letter(s) above the bars indicate no significant differences of values in measured parameters at *p* < 0.05. C—control, non-diabetic rats; DM—control, type 1 diabetic rats; CHR506—type 1 diabetic rats treated by gavage with chrysin at a dose of 50 mg/kg for 28 days; CHR100—type 1 diabetic rats treated by gavage with chrysin at a dose of 100 mg/kg for 28 days; TOS—total oxidative status; TAR—total antioxidant response; OSI—oxidative stress index.

There were no significant changes in the total oxidative status (TOS) and total antioxidant response (TAR) in the lenses of the DM rats when compared with the C rats, but the oxidative stress index (OSI), calculated based on the TOS and TAR values, was significantly higher in the lenses of the DM rats than in the lenses of the C rats. TOS values in the lenses of both chrysin-treated rat groups were significantly lower than in the lenses of the DM rats and the C rats. The TAR values in the lenses of the CHR50 and CHR100 rats were significantly higher than in the lenses of the DM rats, and after treatment with the 100 mg/kg dose of chrysin, TAR value significantly exceeded even the values recorded in the lenses of the C rats. As a consequence of the changes in the TOS and TAR after the administration of chrysin to the diabetic rats, the OSI values in the lenses of the CHR50 and CHR100 rats were significantly lower than in the lenses of the DM and C rats (Figure 4).

### 3.5. Effect of Chrysin on Polyol Pathway Andacvanced Glycation End-Products

The induction of diabetes resulted in a significant increase in the lens level of all sugars which are processed in the polyol pathway—glucose, sorbitol and fructose. Moreover, the activity of the enzymes involved in the conversion of these sugars, aldose reductase (AR) and sorbitol dehydrogenase (SDH), was significantly higher in the lenses of the DM rats than in the lenses of the C rats. Chrysin administered to the diabetic rats at doses of 50 and 100 mg/kg did not change the level of the analyzed sugars in the lenses, as compared to the DM rats. However, after administration of the lower dose of chrysin (50 mg/kg), the sorbitol level in the lenses of the diabetic rats was lower than in the lenses of diabetic rats treated with 100 mg/kg of chrysin. Neither of the chrysin doses affected the AR activity in the lenses of diabetic rats. As far as SDH is concerned, its activity was lowered only by the higher dose of chrysin, compared to the DM rats. The advanced glycation end-products (AGEs) level in the lenses of the DM rats was significantly higher than in the C rats and treatment with chrysin did not alter this parameter in comparison with the DM rats (Table 3).

### 3.6. Principal Component Analysis

**Figure 5:**
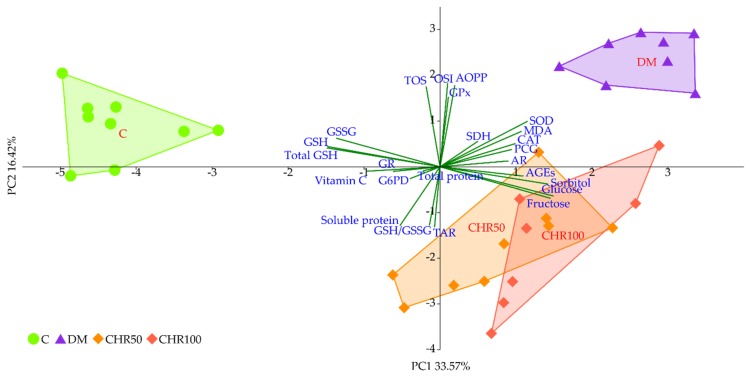
PCA plot of biochemical parameters linked to oxidative stress and polyol pathway measured in the lenses. Red fonts indicate group names: C—control, non-diabetic rats; DM—control, type 1 diabetic rats; CHR50—type 1 diabetic rats treated by gavage with chrysin at a dose of 50 mg/kg for 28 days; CHR100—type 1 diabetic rats treated by gavage with chrysin at a dose of 100 mg/kg for 28 days; blue fonts indicate individual parameters in the lenses; dark green lines represent correlations for individual parameters with regard to principal components (PC1 and PC2).

The PCA revealed a strong separation of the C and DM groups which occurred along principal component 1 (PC1), explaining more than 33% of total variability. The DM rats separated sharply to the right of the plot, whereas the C rats separated to the left. Both CHR50 and CHR100 rat groups remained in between C and DM clusters, near the plot center, however slightly offset to the right. This separation was significant with regard to both the C and DM rat groups, but there was no difference between CHR50 and CHR100. Additionally, both CHR50 and CHR100-derived samples clustered at the bottom half of the plot, whereas C and DM were placed above (separation with regard to PC2). The separation along the PC1 axis was mainly affected by glucose, fructose, sorbitol, AGEs, MDA, SOD and CAT, clearly correlating with the clusters located to the right of the plot (diabetic groups), whereas total GSH, GSH, GSSG and vitamin C correlated more with the cluster located to the left, corresponding to the C rats (Figure 5).

## 4. Discussion

Streptozotocin (STZ; 2-deoxy-2-(3-methyl-3-nitrosourea)-1-d-glucopyranose) is a toxic glucose analog isolated from *Streptomyces achromogenes*. In high doses, STZ is widely used to develop type 1 diabetes in animal research. It accumulates preferentially in pancreatic beta cells and leads to their necrosis. As a result of beta cells death, insulin-dependent diabetes develops in animals. In this model, diabetes is characterized by severe hyperglycemia, polyuria and polydipsia—the symptoms similar to human type 1 diabetes [42,43,44,45].

Hyperglycemia is one of the main reasons for ROS overproduction in diabetic conditions [7]. These oxidants interact with double bonds between carbons in fatty acid chains (especially in polyunsaturated fatty acids) leading to lipid peroxidation and the generation of toxic secondary metabolites, such as malondialdehyde (MDA) [46]. In the lenses, oxidation of lipids may cause the membrane stiffness and, in consequence, light scattering [47]. As we observed in our study, the oxidative stress index (OSI) was significantly higher in the lenses of diabetic rats than in the lenses of healthy control rats, indicating that there was an excess of oxidants in the system. We also noted that in the lenses of the DM rats, MDA level was elevated, which may be a result of the oxidant/antioxidant imbalance. Our results overlap with other studies in which an increase in the MDA level was described in the lenses of diabetic animals [48,49,50,51]. ROS and free radicals may also interact directly with the thiol groups of amino acids in peptides and proteins, which promotes oxidation of these compounds and their structure and/or function change, and may lead to protein aggregation [52]. In the eye lens, there are soluble proteins—crystallins—which ensure lens transparency, since they do not scatter light. The aggregation of these soluble proteins eventually leads to cataract formation [53]. Such aggregates may be formed by an increase in disulfides and disulfide cross-links, the level of which can be measured indirectly by assaying the advanced oxidation protein products (AOPP) [52,54]. During oxidative stress and in the presence of reducing sugars, amino acids may transform to highly reactive carbonyl groups (PCG), which are a good marker for protein oxidative damage in the cells [55,56,57]. As we observed in this study, in the lenses of diabetic rats there were no changes in the total protein level, but the level of soluble protein decreased, while the level of both AOPP and PCG increased. This suggests that, due to the oxidative stress, protein aggregates were formed in the lenses of these animals. Similar results were obtained by other authors [51,58,59]. 

**Figure A1:**
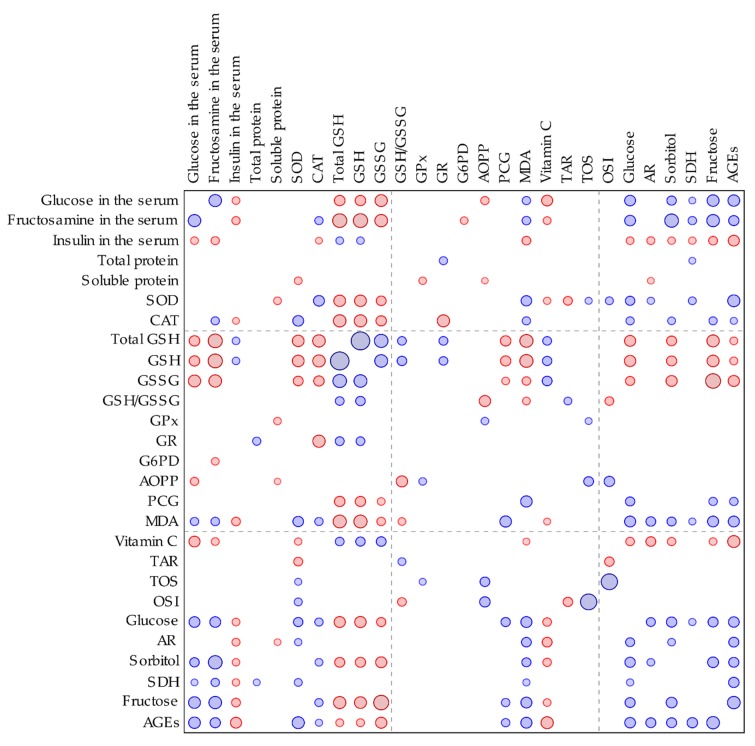
Spearman’s rs correlation plot of biochemical parameters linked to oxidative stress and polyol pathway measured in the lenses, as well as glycemia-related parameters in the serum; blue circles indicate positive correlations, red circles indicate negative correlations, at *p* < 0.05; circles’ sizes correspond to correlation strength (the bigger the circle, the stronger the correlation).

The main non-enzymatic antioxidant responsible for ROS scavenging in the lenses is glutathione (GSH) [60]. In our study, the total and reduced GSH levels in the lenses of diabetic rats were significantly lower than in the lenses of the healthy animals. These parameters correlated negatively with the serological glucose level and the fructosamine level—a parameter representing long-term glycemia (Appendix A, Figure A1).

GSH decrease in the lenses of diabetic rats is widely described in scientific literature [48,49,51,61]. This effect is presumably a response to ROS generation by the elevated serological glucose level. In oxidative stress conditions, the GSH serves as a cofactor for antioxidative enzymes and is oxidized to GSSG. As a result, the GSSG level increases and the GSH/GSSG ratio consequently decreases [62]. In our study, the GSSG level did not increase in the lenses of diabetic rats, but similarly to GSH level, was lowered instead. For this reason, the GSH/GSSG ratio did not change. This effect may be explained by a compensatory mechanism in the lens, in which the excess of GSSG is removed from the lens by the trabecular meshwork in order to maintain the GSH/GSSG ratio [63]. A decrease in the total GSH level in the lenses of diabetic animals resulted from a decrease of both the reduced and oxidized forms of glutathione.

We observed that the GSH level in the lenses of diabetic rats treated with both the doses of chrysin was elevated in comparison to the non-treated rats. This increase, although statistically significant, was minor. Even though, from a mathematical point of view, the GSH/GSSG ratio in the lenses of chrysin-treated diabetic rats was restored to the values observed in the healthy control rats, it should be noted that the total GSH level remained unchanged after chrysin treatment. The main effect observed in the PCA shows a strong negative link between total GSH, GSH and GSSG levels and both chrysin-treated rat groups, suggesting low values in these groups and high values in the control rats. CHR50 and CHR100 rat groups clustered closer to the healthy C rats along PC1, but only ever so slightly. The observed statistically significant increase in reduced GSH level after chrysin administration is a probable result of a completely different mechanism. The PCA provides a clue supporting this reasoning as chrysin-treated rats separate from the remaining clusters also with regard to PC2. This statistically significant effect could not simply be explained by counteracting the diabetic state. Usually GSH is synthetized in the lens epithelial cells and outer cortex, but can also be transported from aqueous humor or be restored from GSSG [63,64]. However, GSH synthesis may also be promoted by flavonoid supplementation. Moskaug et al. provided evidence on the de novo GSH synthesis—the cellular GSH level is then increased via activating gamma-glutamylcysteine synthetase induced by polyphenols [65]. Since in our study the activity of glutathione reductase was not altered by chrysin supplementation, we presume that chrysin promotes de novo GSH synthesis rather than the restoration of GSH from GSSG. Chrysin is reported to increase the GSH level in various organs of diabetic animals [27,66], which is consistent with our report. 

MDA formed during lipid peroxidation can be metabolized by enzymes using GSH as a cofactor to various metabolites which are removed with urine [46]. GSH is also a crucial molecule in the prevention of protein oxidation. GSH may form a reversible disulfide bond with proteins, which protects these molecules (including crystallins) from misfolding and aggregation [52,53,67]. Therefore, the increased GSH level in the lenses of the diabetic rats treated with chrysin (especially the 50 mg/kg dose) may be connected with increased soluble protein level and decreased levels of protein and lipid oxidation markers. Similarly to our in vivo experiment, in the ex vivo study conducted on the goat lenses incubated in the medium with a high concentration of glucose, chrysin elevated the soluble protein level and decreased the PCG level [20]. In our previous study, we observed that administration of another flavone—diosmin—resulted in the AOPP level decrease in the lenses of diabetic rats [29]. Treatment with other natural-derived compounds or plant extracts resulted in the MDA and PCG levels’ decrease in the lenses of diabetic rats, in comparison to the untreated diabetic animals [49,51,59,68], which overlaps with our results. In the study conducted ex vivo on the rat lenses, chrysin at a concentration of 200 µM added to the medium: (1) simultaneously with sodium selenite, (2) as a pretreatment before sodium selenite, or (3) after a 30-minute long preincubation with this salt, reduced the MDA level and elevated the GSH level [21]. 

Antioxidative enzymes, such as superoxide dismutase (SOD), catalase (CAT) and glutathione peroxidase (GPx), are involved in the antioxidative defense of the organism [69]. Their activity is regulated via nuclear factor erythroid 2–related factor 2 (Nrf2) dependent pathway. In normal non-stressed conditions, the Nrf2 is connected to its cytoplasmatic inhibitor—Kelch-like ECH-associated protein-1 (Keap1). When oxidative stress increases, Nrf2 is released from Keap1, then translocated to the nucleus where it connects with antioxidant response element (ARE), inducing the expression of Nrf2-ARE dependent genes, including the genes coding antioxidative enzymes [70]. We observed that SOD and CAT activity was significantly increased in the lenses of diabetic rats. This elevated activity may be connected to the fact that oxidative stress in these lenses was observed, based on the OSI value. We did not note an increase in the GPx activity. GPx possesses a very high affinity to H_2_O_2_ and eliminates it even when the latter is in low concentration. However, this process requires electron donors such as GSH, therefore is disadvantageous for the cell. CAT has a lower affinity to hydrogen peroxide but a very high Michaelis constant (K_M_) to the substrate, thus may eliminate H_2_O_2_ even at very high concentrations [71]. We hypothesize that GPx activity in the lenses was not altered by diabetes, since GSH was utilized to form S-conjugates with proteins in order to protect them against oxidative stress, and its resources had depleted. After chrysin administration at both the doses, the activity of SOD and CAT decreased. This may be a result of the overall reduction of oxidative stress in the lenses. The OSI parameter was significantly reduced in the lenses of diabetic rats treated with 50 and 100 mg/kg of chrysin. As OSI correlates with SOD, its decrease would result in the SOD activity reduction. When SOD does not dismutate the superoxide radical, there is no need to activate CAT which is also positively correlated with SOD (Appendix A, Figure A1). This may suggest that chrysin, being an exogenous antioxidant, administered to the diabetic rats, scavenged ROS, and thus reduced oxidative stress in the lenses (lowered OSI), which led to the decrease of Nrf2-ARE dependent antioxidative gene expression.

Glucose is transported to the lens in an insulin-independent process. In normal physiological conditions, glucose in the lenses is metabolized mainly by the hexokinase-involving pathway, but in a hyperglycemic state the hexokinases become saturated and the polyol pathway takes the leading role in the lenticular glucose metabolism [11,72]. As we observed in our study, the non-fasting glucose level in the serum as well as the fructosamine level were considerably elevated in comparison with the healthy, non-diabetic rats. These elevated glycemia-related markers correlated with the increased markers connected to the polyol pathway in the lenses (Appendix A, Figure A1). In other studies concerning the lenses of the diabetic animals, the polyol pathway was also intensified and markers describing the sugars and enzymes connected to this pathway were elevated [51,61,73]. Administration of chrysin did not reduce the non-fasting glucose level in the serum of the tested rats. This was confirmed by a marker representing a long-term glycemia state—fructosamine, which was not altered after 28 days of chrysin treatment. Lack of changes in the glycemia-related markers in the serum of the rats treated with chrysin may explain why the polyol pathway was not inhibited in the lenses. Some studies indicate that chrysin has no effect on hyperglycemia in the rats [74,75], which is consistent with our study. An increase in the advanced glycation-end products (AGEs) level is a result of an intensified polyol pathway and fructose accumulation in the lenses [76]. We observed that in the lenses of the diabetic animals, the AGEs level was significantly higher than in the non-diabetic rats. The AGEs level correlates positively with the polyol pathway, serological glucose and fructosamine levels (Appendix A, Figure A1). Since the polyol pathway in the lenses and serological glucose level were not altered by chrysin administration, the AGEs level in the lenses also remained unchanged. AGEs interact with the specific receptors—RAGE (receptor of AGE). As a result of this interaction, ROS are generated through the activation of NADPH oxidase. Therefore, the excess of AGEs results in oxidative stress progression [77,78]. We noted that the AGEs level in the lenses positively correlates with the SOD activity in this organ (Appendix A, Figure A1), indicating that increased SOD activity is a response to overly produced ROS, generated by AGEs. Moreover, AGEs level correlates negatively with GSH and vitamin C levels in the lenses (Appendix A, Figure A1), which also suggests a compensative mechanism for ROS removal from the lens by small-molecule antioxidants. These non-enzymatic antioxidants (GSH and vitamin C) are restored from their oxidized forms by enzymes requiring NADPH—glutathione reductase and thioredoxin reductases, respectively. Moreover, synthesis of vitamin C requires NADPH as a driving force for the enzymes involved in this process [79,80]. Aldose reductase (AR), a first enzyme in the polyol pathway catalyzing the conversion of glucose to sorbitol, competes for NADPH with GR [76]. It is possible, that in hyperglycemic condition, AR utilizes most of the NADPH supplies in the cell, thus GSH cannot be restored from GSSG. AR activity in the lenses negatively correlates with the vitamin C level in this organ (Appendix A, Figure A1), which was also confirmed by the PCA. This suggests that due to the lack of NADPH, vitamin C cannot be reduced from the oxidized forms or be synthetized de novo in the rat lenses. In our study, AR activity was not affected by chrysin administration. On the contrary, in the ex vivo study, chrysin revealed a strong AR inhibitory effect [20]. However, it should be noted that in the ex vivo experiment, chrysin was added to the medium in which lenses were incubated, thus the lenses were exposed directly to this flavonoid action. In the in vivo experiments, when substances are given orally or by intragastric tube, they may be metabolized or bound to plasma proteins and, in consequence, they do not interact directly with the organ [81,82]. Therefore, the discrepancies between our investigation and the ex vivo study are a result of experimental design and the complexity of the living organism.

In order to evaluate the significance of the dose-dependent effect of chrysin on the lenses of diabetic rats, the PCA was performed. According to this analysis, no differences were observed between 50 and 100 mg/kg doses. Due to the composite nature of PCA, only the most prominent differences regarding individual parameters are exposed. This reveals itself in different weights represented by variable correlation strengths with regard to the PC1 and PC2 axes, along which the experimental group clusters separate from each other, thus providing insight into the significance of each parameter to the total observed variance [83]. This phenomenon explains the inconsistencies observed between measurements of the individual parameters, providing only a quantitative aspect when compared to the effect revealed by the PCA, where all parameters are considered simultaneously in both quantitative and qualitative manner.

**Figure 6:**
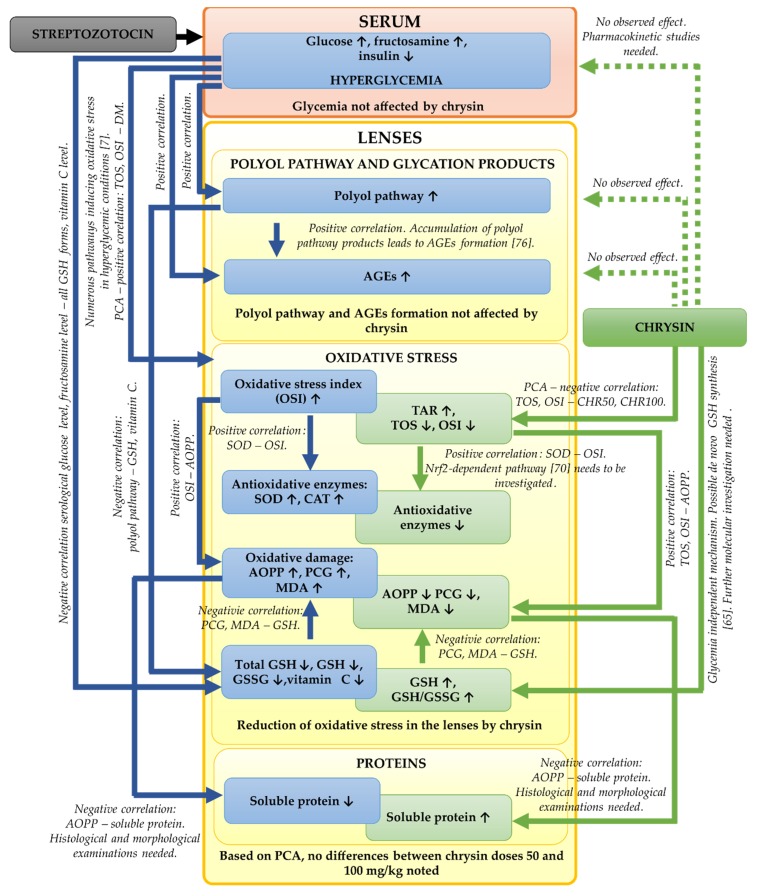
Graphical presentation of the effects observed in the present study. Suggested steps of future studies are also indicated in the appropriate places. ↑—increase in parameter; ↓—decrease in parameter; blue boxes connected by blue arrows—effects observed in the DM rats; green boxes connected by green arrows—effects observed in the chrysin-treated diabetic rats; green dotted arrows—no effect observed after chrysin administration to the diabetic rats.

In the presented study, we focused mainly on the biochemical parameters related to the oxidative stress and polyol pathway in the lenses of the diabetic rats treated with chrysin. It should be noted that based on our results, further, more detailed examinations are needed. We did not examine the histological or morphological changes in these lenses. Such examinations should be assessed in the future in order to validate the biochemical results. Moreover, to explain molecular mechanisms behind the observed biochemical changes (e.g., the increase of GSH level in the lenses), genetic assays are crucial. The bioavailability of chrysin in the diabetic animals can be altered in comparison to the healthy rats, therefore pharmacokinetic studies regarding chrysin administered to the healthy and diabetic rats at doses 50 and 100 mg/kg could be informative. The effects observed during the study including further steps required in future experiments are presented in a combined form in Figure 6.

## 5. Conclusions

In this study we demonstrated that chrysin administered by gavage for 28 days reduced oxidative stress in the lenses of the male type 1 diabetic rats by decreasing the activity of antioxidative enzymes, reducing the levels of the oxidative damage markers and improving the oxidative stress index. The administration of chrysin also slightly increased the reduced glutathione level in the tested organ. However, chrysin did not improve the glycemia-related parameters in the serum and, as a consequence, did not alter the intensified polyol pathway or prevent AGEs formation in the lenses of diabetic rats. Although the individual ANOVAs revealed differences in the effect on some parameters between the used doses, the PCA of all tested lens parameters showed that the effect of the 50 mg/kg dose did not differ from the 100 mg/kg dose. Thus, in further studies regarding the effect of chrysin on the eye lens of diabetic subjects, the dose of 50 mg/kg can be considered sufficient to obtain a beneficial pharmacological effect. Moreover, this lower dose reduced all the markers linked to oxidative damage in the lenses. Based on the multivariate analysis performed in the study, we suppose that chrysin reveals an additional metabolic effect not connected with the diabetic state. Therefore, obtained results lead to the conclusion that chrysin has antioxidative activity in the lenses of diabetic rats and minimizes the oxidative damage but is not an antihyperglycemic agent. After more detailed studies, chrysin may be considered only as a supplementary compound used by diabetics in order to prevent the oxidative stress-derived changes in the lenses. 

## Figures and Tables

**Table 1:** Effect of chrysin on glycemia-related markers in the serum of type 1 diabetic rats.

Parameter	C	DM	CHR50	CHR100
Glucose (mg/dL)	141.35 ± 32.97 ^c^	641.85 ± 80.93 ^ab^	687.91 ± 64.54 ^a^	588.84 ± 142.35 ^b^
Fructosamine (µmol/L)	281.86 ± 29.49 ^b^	495.56 ± 65.20 ^a^	483.27 ± 97.48 ^a^	444.36 ± 62.64 ^a^
Insulin (µg/L)	0.434 ± 0.272 ^a^	0.089 ± 0.053 ^b^	0.171 ± 0.136 ^b^	0.136 ± 0.152 ^b^

Results are presented as arithmetical means ± SD. The letters in the superscripts indicate statistical significances (one-way ANOVA followed by Fisher’s LSD test). The same letter(s) (a–c) in individual rows indicate no significant differences of values in measured parameters at *p* < 0.05; C—control, non-diabetic rats; DM—control, type 1 diabetic rats; CHR50 – type 1 diabetic rats treated by gavage with chrysin at a dose of 50 mg/kg for 28 days; CHR100—type 1 diabetic rats treated by gavage with chrysin at a dose of 100 mg/kg for 28 days.

**Table 2:** Effect of chrysin on glutathione-related enzymes’ activity, protein level and vitamin C level in the lenses of type 1 diabetic rats.

Parameter	C	DM	CHR50	CHR100
GPx (nmol/min/mg of protein)	1.19 ± 0.05 ^a^	1.24 ± 0.13 ^a^	1.06 ± 0.12 ^b^	1.17 ± 0.10 ^ab^
GR (nmol/min/mg of protein)	0.51 ± 0.19 ^NS^	0.33 ± 0.10 ^NS^	0.48 ± 0.22 ^NS^	0.32 ± 0.13 ^NS^
G6PD (U/mg of protein)	0.040 ± 0.006 ^NS^	0.029 ± 0.011 ^NS^	0.037 ± 0.017 ^NS^	0.035 ± 0.012 ^NS^
Total protein (mg/g of the lens)	563.2 ± 99.7 ^NS^	536.4 ± 123.5 ^NS^	560.5 ± 58.8 ^NS^	562.3 ± 187.9 ^NS^
Soluble protein (mg/g of the lens)	443.8 ± 25.2 ^a^	408.5 ± 39.2 ^b^	451.3 ± 13.7 ^a^	445.0 ± 19.7 ^a^
Vitamin C (µg/g of the lens)	7.69 ± 0.14 ^a^	7.17 ± 0.06 ^b^	7.27 ± 0.55 ^b^	7.40 ± 0.14 ^ab^

Results are presented as arithmetical means ± SD. The letters in the superscripts indicate statistical significances (one-way ANOVA followed by Fisher’s LSD test). The same letter(s) (a,b) in individual rows indicate no significant differences of values in measured parameters at *p* < 0.05; NS—not significant. C—control, non-diabetic rats; DM—control, type 1 diabetic rats; CHR50—type 1 diabetic rats treated by gavage with chrysin at a dose of 50 mg/kg for 28 days; CHR100—type 1 diabetic rats treated by gavage with chrysin at a dose of 100 mg/kg for 28 days; GPx—glutathione peroxidase; GR—glutathione reductase; G6PD—glucose-6-phosphate dehydrogenase.

**Table 3:** Effect of chrysin on polyol pathway markers and advanced glycation end-products level in the lenses of type 1 diabetic rats.

Parameter	C	DM	CHR50	CHR100
Glucose (µmol/g of the lens)	0.9 ± 0.6 ^b^	4.6 ± 1.1 ^a^	4.2 ± 1.1 ^a^	4.5 ± 0.5 ^a^
AR (nmol/min/mg of protein)	0.09 ± 0.01 ^b^	0.11 ± 0.02 ^a^	0.10 ± 0.02 ^a^	0.11 ± 0.02 ^a^
Sorbitol (µmol/g of the lens)	1.2 ± 0.1 ^c^	30.6 ± 1.8 ^ab^	29.7 ± 1.7 ^b^	32.4 ± 2.7 ^a^
SDH (µU/mg of protein)	2.0 ± 0.5 ^b^	2.9 ± 0.9 ^a^	2.8 ± 0.9^a^	1.9 ± 0.7 ^b^
Fructose (µmol/g of the lens)	0.076 ± 0.002 ^b^	0.135 ± 0.007 ^a^	0.138 ± 0.005 ^a^	0.137 ± 0.009 ^a^
AGEs (µg/g of the lens)	7.47 ± 1.88 ^b^	10.83 ± 0.49 ^a^	11.12 ± 0.89 ^a^	10.66 ± 1.25 ^a^

Results are presented as arithmetical means ± SD. The letters in the superscripts indicate statistical significances (one-way ANOVA followed by Fisher’s LSD test). The same letter(s) (a–c) in individual rows indicate no significant differences of values in measured parameters at *p* < 0.05. C—control, non-diabetic rats; DM—control, type 1 diabetic rats; CHR50—type 1 diabetic rats treated by gavage with chrysin at a dose of 50 mg/kg for 28 days; CHR100—type 1 diabetic rats treated by gavage with chrysin at a dose of 100 mg/kg for 28 days; AR—aldose reductase; SDH—sorbitol dehydrogenase; AGEs—advanced oxidation end-products.

## Appendix B

MANOVA–PCA Score Coordinates (Axes Combined)

Effect	Test	Value	F	Effect df	Error df	*p*
Group	Wilk	0.011210	78.81756	6	56	0.000000

PCA Axes Individually

Effect	df	PC1				PC2			
		SS	MS	F	*p*	SS	MS	F	*p*
Group	3	239.2633	79.75444	124.7430	0.000000	99.3275	33.10915	35.87145	0.000000
Error	29	18.5412	0.63935			26.7668	0.92299		
Total	32	257.8045				126.0943			

LSD Post-Hoc Axis 1

Group	PC1 Mean	b	c	a
C	−4.25372		****	
CHR50	0.76779	****		
CHR100	1.44477	****		
DM	2.65751			****
**** *p* < 0.05.				

LSD Post-Hoc Axis 2

Group	PC2 Mean	c	b	a
CHR50	−1.73981	****		
CHR100	−1.64441	****		
C	0.88476		****	
DM	2.40081			****
**** *p* < 0.05.				
